# Novel coronavirus pneumonia (COVID-19) combined with Chinese and Western medicine based on ”Internal and External Relieving -Truncated Torsion” strategy

**DOI:** 10.1097/MD.0000000000023874

**Published:** 2020-12-18

**Authors:** Xinxin Wu, Wei Lu, Quan Guo, Min Cao, Wen Zhang, Changya Liu, Yao Qu, Wei Peng, Jinhua Che, Bangjiang Fang, Shuang Zhou

**Affiliations:** a LongHua Hospital Shanghai University of Traditional Chinese Medicine, NO.725 Wanping South Road, Xuhui District; b Shanghai University of Traditional Chinese Medicine, NO.1200 Cai Lun Road, Zhangjiang Hi-Tech Park, Pudong New Area, Shanghai; c Hubei Provincial Hospital of TCM, NO.856 Luoyu Road, Hongshan District, Wuhan, China.

**Keywords:** COVID-19, exterior and interior solutions, prospective cohort study, traditional Chinese medicine, truncated torsion

## Abstract

**Introduction:**

The outbreak of novel coronavirus disease 2019 (COVID-19) has caused a global pandemic since December 2020. It has not only associated with physiological disorder but also with psychological distress and symptoms of mental illness. Whether the vaccines and antivirals can provide protects remains unknown. Traditional Chinese medicine (TCM) is recommended as an alternative and significant way of preventing and treating COVID-19 in China. However, few studies have assessed the benefits of this treatment and mental health after they recover. Our study is designed to investigate effects and safety when using TCM on the course of this disease and the impact of COVID-19 on pandemic-related anxiety.

**Method:**

For this prospective cohort study, we will enroll 300 COVID-19 patients aged 18 to 80 years at 4 centers. We divide them into 2 groups, according to whether they use *Baidu Jieduan Granule* at a ratio of 1:1. We will compare treatments combined *Baidu Jieduan Granule* with conventional Western medicine (experimental group) vs treatment of conventional Western medicine only (control group). The basic information of patients including demographic, general condition, primary diseases, and complications will be assessed. Related examines will be conducted at 1, 3, 5, 7, and 14 days. The primary outcomes are clinical outcome. A follow-up time of 1 year (to June 30, 2021) allow us to evaluate the psychiatric disorder after recovery. We will monitor adverse events throughout the trial.

**Discussion:**

It will be the first prospective cohort study which uses *Baidu Jieduan Granule*, based on the innovation traditional Chinese medicine strategy of “ Internal and External Relieving -Truncated Torsion ” to treat the common type of COVID-19. The result of this study may provide evidence-based recommendations of TCM for treatment and psychological distress or symptoms of mental illness of the common type of COVID-19.

## 1 Introduction

Since December 2019, patients with pneumonia-like symptoms of unknown origin was found 1 after other in Wuhan city, Hubei Province.^[1,2]^ Soon after the outbreak, researchers obtained the whole genome sequence by sequencing from the patients samples, and the result revealed that it was a novel coronavirus (COVID-19).^[1]^ The International Committee on Taxonomy of Viruses (ICTV) named the virus as severe acute respiratory syndrome coronavirus 2 (SARS-CoV-2). This kind of virus spread quickly between humans, leading to a worldwide pandemic. As of May 20, 2020, more than 4.7 million people across 215 countries or territories have been infected, of which more than 316 000 cases died.^[3]^ In February 2020, the WHO (World Health Organization) declared the outbreak as the public health emergency of international concern (PHEIC) for the situation getting worse and worse.^[4]^ Therefore, how to prevent and cure this kind of new infectious disease is of high significance.

Analyzing some retrospective studies of COVID -19 cases,^[5,6]^ the commonest manifestations were respiratory and digestive symptoms. Those severe patients could rapidly progress to acute respiratory distress syndrome, septic shock, refractory metabolic acidosis, and coagulation dysfunction, resulting in life-threatening. Currently, there is no specific medicine for the treatment of COVID-19. As we know, most antiviral drugs undergoing clinical testing in patients with COVID-19 are originally developed against influenza, HIV, Ebola, SARS, and MERS.^[7,8]^ But their effects and safety remain unknown. Furthermore, some findings suggest that psychiatric complications for people affected by COVID-19 are universal including post-traumatic stress symptoms (PTSS)/post-traumatic stress disorder (PTSD), anxiety and depression. Experts point out the need to pay specific attention to them at risk of further distress that may need tailored interventions.^[9]^

“Internal and External Relieving -Truncated Torsion” strategy means in the very beginning of COVID-19, we use TCM which can release the exterior (sweating) or dredging the interior (draining and defecating) to intervene timely so that we can remove pathogenic factors, treat diseases and then prevent common type COVID-19 patients from developing into severe and critical illnesses. This theory originates from the idea of “ preventive treatment of disease” in “ *Inner Canon of Yellow Emperor*”. In the long-term struggling against hundreds of thousands of times of infectious disease outbreak, this theory gradually got improved and perfected. Wu Youke, the most famous expert of contagious disease in Ming Dynasty, proposed that the pathogenic factors should be eliminated as soon as possible in his book “*Plague Theory*”.^[10]^ Ye Tianshi, another great medical expert, also put forward the viewpoint of protecting the organs which are not attacked first in “*Treatise on Epidemic Febrile Diseases*”.^[11]^

The main components of *Baidu Jieduan Granule* are *Cmnamomi Mmulus* which can promote sweating, *Radix Rhei Et Rhizome* and *Gypsum Fibrosum* both of which can dredge the interior. We also add some other components with heat-clearing and antiviral effects considering the features of COVID-19. Among them, *Cmnamomi Mmulus*, *Radix Rhei Et Rhizome* and *Gypsum Fibrosum* have shown sound clinical results in the treatment of viral and bacterial pneumonia for more than 10 years.^[12,13]^ Since January 2020, *Baidu Jieduan Granule* has been applying in hospitals in Wuhan and other epidemic areas in Hubei province to cure COVID-19 or some suspicious patients with respiratory infection symptoms. Combining the data from these studies and data from previous, it demonstrated that *Baidu Jieduan Granule* is of great effects and high safety. Moreover, modern pharmacological studies have proved that *Radix Rhei Et Rhizome* has the function of antibacterial and antivirus, for example, streptococcus A, streptococcus B, Streptococcus pneumonia, staphylococcus aureus, influenza virus A and influenza virus B. It can also significantly reduce the levels of TNF-α, IL-2, IL-6, IL-8 and other inflammatory cytokines in patients with systemic inflammatory response syndrome (SIRS), inhibit the excessive inflammatory reaction and maintain immune stability.^[14]^ In primary studies, *Cmnamomi Mmulus* plays an immunosuppressive role by inhibiting the proliferation of spleen cells.^[15]^ Cinnamyl alcohol can alleviate airway and lung inflammation by reducing the release of inflammatory factors and cytokines.^[16]^*Gypsum Fibrosum* is composed of 2 layers of silica tetrahedron and 1 layer of magnesium oxide octahedron.^[17]^ The surface adsorption capacity and electrostatic adsorption capacity of *Gypsum Fibrosum* can fix the virus and its toxin on the mucosal surface of the small intestine, making it lose the ability to cause disease.^[18,19]^

In this multicenter, prospective cohort study, we aim to use the *Baidu Jieduan Granule* combined with Western medicine to dynamically observe the changes of patient's blood, cytokines, coagulation function, chest computed tomography (CT), respiratory distress (respiratory rate, RR), blood oxygen saturation, oxygenation index, and patient prognosis (healing, worsening, and death),to prove the clinical efficacy of *Baidu Jieduan Granule* in curing COVID-19, to reveal the possible mechanism from the immune route, and to evaluate the safety. We will also assess mental health outcomes through our 1 year follow-up.

## 2 Methods and designs

### 2.1 Study design and settings

This research is designed as a multicenter prospective cohort study conducted at 4 medical centers, including Huangshi Hospital of TCM (Huangshi City, Hubei Province), LaoHeKou Hospital of TCM (LaoHekou City, Hubei Province), Hubei Provincial Hospital of TCM (Wuhan City, Hubei Province) and LongHua Hospital Shanghai University of TCM (Shanghai City, Shanghai). LongHua Hospital Shanghai University of TCM is the leading medical center. These 4 TCM hospitals are the main medical institutions responsible for the treatment of COVID-19 patients. In order to strengthen the cooperation, we will establish a coordination center. This center contains medical experts, statisticians and quality control experts from other hospitals and universities. The primary function of this center is to provide vital clinical decisions or recommendations, conduct data statistic, monitor the research process.

### 2.2 Objectives

The main aim of our trail is to investigate the efficacy and safety of *Baidu Jieduan Granule* in the common type of COVID-19 patients and assess mental health outcomes through our 1year follow-up.

### 2.3 Population

**Figure 1:**
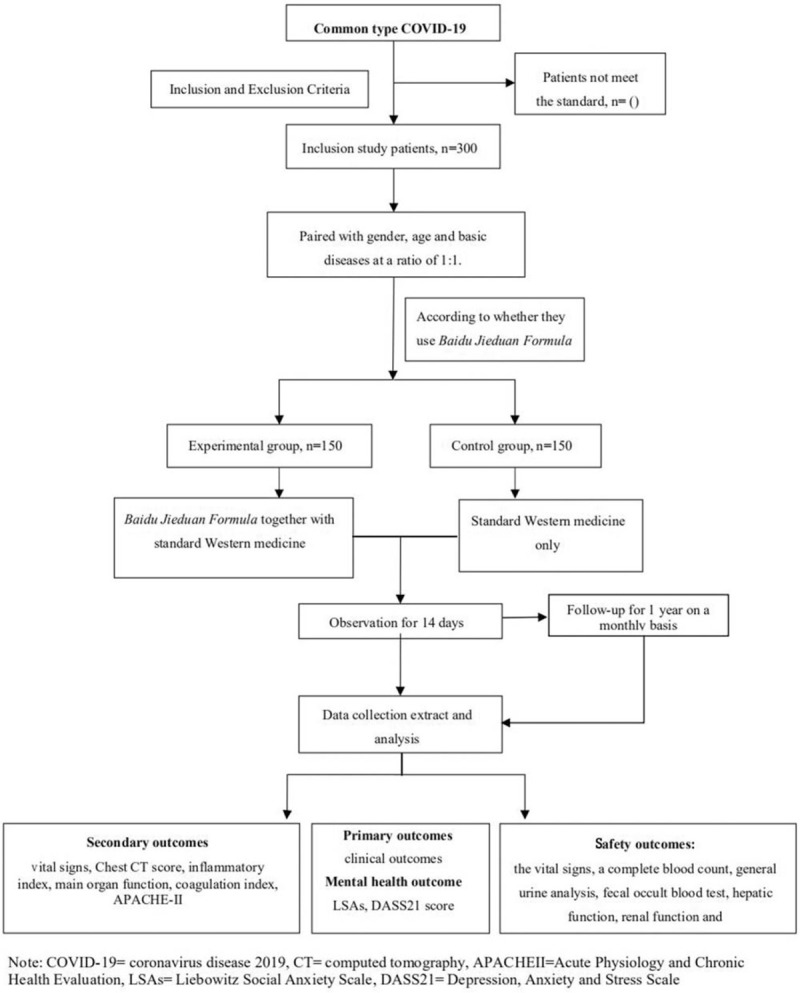
Flowchart of study design.

For this multicenter prospective cohort study, we will enroll 300 COVID-19 patients meeting the inclusion criteria from 4 medical centers. The recruitment duration will be lasted from February 2020 to June 2020; the follow-up will end on June 30, 2021. The study flowchart is as Figure 1.

### 2.4 Inclusion criteria

1. Patients diagnosed with COVID-19^[20]^
  1) Meeting one of the following evidences:
    1. Real-time reverse transcription-polymerase chain (RT-PCR) detection of new coronavirus nucleic acid shows positive.
    2. The tested virus gene is highly homologous to SARS-CoV-2.
    3. Serum SARS-CoV-2 lgM and lgG antibody are positive.
    4. Serum SARS-CoV-2 IgG antibody turns positive or the serum novel coronavirus specific IgG antibody in the recovery phase is 4 times or higher than that in the acute phase.
  2) Patient has the symptoms of cough, expectoration, chest pain, dyspnea, and fever.
  3) Imaging findings of pneumonia.
2. 18 years old ≤ age ≤80 years old;
3. Informed consent, participate in this study voluntarily.

### 2.5 Exclusion criteria

1. The patient was allergic to *Baidu Jieduan Granule.*
2. Pregnant and lactating women.
3. Patients with obstructive pneumonia, pulmonary interstitial fibrosis, pulmonary alveolar proteinosis and allergic alveolitis caused by lung tumor
4. Patients with a malignant tumor, liver cirrhosis, chronic renal failure (uremic stage), hematological disease, HIV, and other serious diseases.
5. Patients who were treated with hormones and immunosuppressant for a long time.
6. Patients who suffer from severe mental illness or are unable to cooperate with this experiment.

### 2.6 Patient withdrawal

1. Subjects experiencing serious adverse events at any time during the treatment period.
2. Subjects giving up the study midway.

### 2.7 Sample size

Considering that COVID-19 is a newly emerging infectious disease, and it is the first time we use *Baidu Jieduan Granule* for the treatment of COVID-19, there are no reference resources at present that we cannot calculate the exact sample size. We suppose that the withdrawal rate is about 10%^[21]^ and about 300 patients that will be enrolled in our study, and an average of 75 patients will be admitted to each medical center. The collected data will provide a basis for calculating sample size in future and facilitate the future research.

### 2.8 Grouping

Three hundred COVID-19 cases aged 18 to 80 years are assigned into 2 groups (experimental group and control group) basing on whether they use *Baidu Jieduan Granule* at a ratio of 1:1. The experimental group and the control group are paired with gender, age and basic diseases so that they are comparable.

### 2.9 Interventions

Patients in the experimental group use *Baidu Jieduan Granule* together with conventional Western medicine, while the control group accept standard Western medicine only. The *Baidu Jieduan Granule* comprises *Scutellariae Radix* (30 g), *Radix Rhei Et Rhizome* (15 g), *Gypsum Fibrosum* (45 g), *Cmnamomi Mmulus* (9 g), *Polygoni Cuspidati Rhizoma Et Radix* (30 g), *Verbenae Herb* (3 g), The *Baidu Jieduan Granule is* brownish yellow particles and provided by Beijing Kangrentang Pharmaceutical Co. LTD (Number: Jing 201800603), which is one of the biggest companies specializing in the production of Chinese medicine Granules.

### 2.10 Outcomes and measurements

#### 2.10.1 Primary outcomes

The primary outcomes of our study are clinical outcomes. In our study, there are 4 kinds of clinical outcomes:

1. recovery, the following conditions shall be met:
  1. the body temperature is normal for 3 consecutive days
  2. respiratory symptoms disappeared completely,
  3. the nucleic acid test of respiratory tract samples is negative for 2 consecutive times (the sampling interval is at least 1 day);
2. condition improved, meet any of the following 2 items:
  1. the clinical symptoms are significantly relieved,
  2. the nucleic acid of sputum, nasopharynx test paper and other respiratory tract samples are negative for 2 consecutive times (the sampling interval should be at least 24 hours);
3. condition worsen, one of the following situations occurs:
  1. shortness of breath, respiratory rate (RR) ≥30 times/minutes,
  2. oxygen saturation ≤93% in resting state,
  3. arterial oxygen partial pressure (PaO2) / fraction of inspired oxygen (FiO2) ≤300 mm Hg,
  4. shock,
  5. respiratory failure, requiring ventilator support,
  6. organ failure needing intensive care, and
  7. death.

We count the number of patients of each kind of clinical outcomes then calculate the ratio.

#### 2.10.2 Second outcomes

1. Vital signs (d1-14), temperature, heart rate, blood pressure, respiratory rate (RR), oxygen saturation.
2. Chest computed tomography (CT) score (d1, d7, d14), in our study we use high resolution computed tomography (HRCT). According to the existing scoring standards,^[22,23]^ each lobe will be graded. 0 points = no pathological changes of lung lobe, 1 point = pathological changes in 0% to 25% of lung lobes, 2 points = pathological changes in 25% to 50% of lung lobes, 3 points = pathological changes in 50% to 75% of lung lobes, 4 points = pathological changes in 75% to 100% of lung lobes, the total score of 5 lobes is used as the chest CT lesion score of each patient. We compare the mean value of lung CT scores.
3. Inflammatory index (d1, d3, d5, d7, d14): peripheral hemogram (the count of peripheral blood leukocyte and lymphocyte), C-reactive protein (CRP), procalcitonin (PCT), tumor necrosis factor (TNF-a), interleukin-6 (IL-6), interleukin-10 (IL-10).
4. Main organ function (d1, d3, d5, d7, d14): alanine aminotransferase (ALT), aspartate aminotransferase (AST), total serum bilirubin (STB), blood urea nitrogen (BUN), serum creatinine (Cr).
5. Coagulation index (d0, d7, d14): blood platelet count (PLT), D-dimer, activated partial thromboplastin time (APTT), prothrombin time (PT), fibrinogen (Fg).
6. Acute Physiology and Chronic Health Evaluation (d1, d3, d5, d7, d14, APACHE-II, Additional file 2) score: we compare the mean value of APECHA II the scores between 2 groups, and calculate improvement rate in APECHA II score = (d14 scored-0 score)/d0 score.

#### 2.10.3 Safety outcomes

The abnormal of vital signs, blood examination, urine examination, liver and kidney function, fecal occult blood, coagulation function and electrocardiogram are recorded in detail and comprehensively analyzed. If they are related to drugs, we will immediately stop using the drug.

#### 2.10.4 Adverse event reporting

Adverse reaction (AE) refers to the occurrence of adverse reactions that are not related to the purpose of treatment in the process of preventing, diagnosing, or treating diseases according to normal usage and dosage. The AE is decided by the clinical expert from the coordination center. The time of onset, clinical manifestations, possible related drugs, solutions, and outcomes should be recorded.

#### 2.10.5 Mental health outcomes

In our study we evaluate psychiatric disorder symptoms of patients with Liebowitz Social Anxiety Scale (LSAs, Additional file 3) and Depression, Anxiety and Stress Scale (DASS21, Additional file 4). LASs measure assesses the way that social phobia plays a role in our life across a variety of situations. There are 24 items in LSAs, each item has 2 questions, the first question asks how anxious or fearful you feel in the situation (0=None, 1=Mild, 2=Moderate, 4=Severe), the second question asks how often you avoid the situation (0=Never, 1=Occasionally, 2=Often, 3=Usually). The final score 55–65 means moderate social phobia, 65–80 marked social phobia, 80–95 severe social phobia, greater than 95 very severe social phobia. DASS21 is a 21 question assessment that was developed to measure the degree of depression, anxiety and stress in an individual. It includes 7 questions for each and the scores are calculated by adding up the scores for each item in the section. There are 5 rating (Normal, Mild, Moderate, Severe, Extremely Severe) of each section according to the score.

### 2.11 Follow-up

After finishing the interventions of 2 weeks, telephone follow-up will be done on all patients for 1 year. We follow these patients on a monthly basis. If the patients were died, we will terminate our follow-up.

### 2.12 Statistical analysis

Statisticians from coordination center will oversee this work. We analysis all data sets by IBM SPSS Statistics 24.0 software. We express collected data by mean ± standard deviation or median and interquartile distance. The comparison between groups of measurement data which accorded with normal distribution and homogeneous variance is *t*-test. At the same time, the nonparametric test is used for non-compliance with a normal distribution. Chi-Squared test will be used to compare the count data or rate, and the logistic test to compare the survival rate between groups. P value <.05 is considered as statistically significant.

### 2.13 Trial management

#### 2.13.1 Coordination center

We will establish a coordination center to ensure that the research goes on wheels. This institution consists medical experts, statisticians, and quality control experts. They are responsible for providing vital clinical decisions or recommendations, coordinating the work of each center, arranging data, conducting data statistic, supervising the research process.

#### 2.13.2 Quality control

Before the study, we will evaluate the 4 hospitals participating in our research and conduct professional training for researchers according to the study protocol. All our work is implemented strictly in accordance with the items of the Clinical Research Organization (CRO).

### 2.14 Data collection and management

We design the case report form (CRF) table as it presented in Additional file 5 by our study protocol. After collecting the information of patients, we will complete the CRFs rightly and timely. Then we input and organize them into an excel sheet. Finally, we will hand it over to statistical experts who will perform statistical analysis. The units of measurement should be unified. We will keep the personal and medical information of all patients strictly confidential. Statistical analysis results will be presented by publishing articles in professional journals or websites after finishing this study. The study procedure and assessments flowchart are as Table 1.

**Table 1:** Study procedure and assessment.

		Observation node					Follow-up phase				
Study period	Visiting items	D1	D3	D5	D7	D14			1 year		
Screening stage	Inclusion criteria	X									
	Signing informed consent	X									
	Exclusion criteria	X									
	Matching and distribution	X									
Observation stage	Administration of study drug	X	X	X	X	X					
	Demographic	X									
	General Condition	X									
	Primary disease	X									
	Complication	X									
	Vital signs	X	X	X	X	X					
	Oxygenation index	X	X	X	X	X					
	Oxygen saturation	X	X	X	X	X					
	Chest CT	X		X		X					
	Hepatic function	X	X	X	X	X					
	Renal function	X	X	X	X	X					
	Cardiac function	X	X	X	X	X					
	Inflammatory indicators	X	X	X	X	X					
	Coagulation function	X	X	X	X	X					
	APACHEII score	X	X	X	X	X					
	Adverse event recording	X	X	X	X	X					
	Drug delivery/taking back/ destruction	X	X	X	X	X					
	Clinical outcomes					X					
	Safety evaluation	X	X	X	X	X					
Follow-up phase	LSAs								X		
	DASS21								X		

### 2.15 Quality control

All researchers taking part in our study will accept strict training following our study protocol and be qualified in the implementation of Good Clinical Practice (GCP) training by the State Food and Drug Administration to ensure the quality of training. Patients involved in our study will be screened according to the inclusion and exclusion criteria. Then we will offer a consent form, covering the patients personal information, therapy information, treatment plans, researches duties and obligations, patients duties and obligations, possible drug-related side effects, etc. Every patient should sign informed consent. *Baidu Jieduan Granule* used in our research are provided by the Beijing Kangrentang Pharmaceutical Co. LTD uniformly to ensure their effects. Our work will be supervised by the coordination center once a month to until we finish our research.

### 2.16 Ethics

This trial is conducted adhering to Helsinki Declaration and Chinese Good Clinical Practice. The Medical Ethics Committee of LongHua Hospital Shanghai University of Traditional Chinese Medicine has approved our study protocol (approval number 2020LCSY007). If there is any change in the research plan or violation of confidentiality regulations, we will submit it to the Ethics Committee for re-examination. We also have registered with the Chinese Clinical Trial Registry (ChiCTR2000030836). http://www.chictr.org.cn/showproj.aspx?proj=51054.

## 3 Discussion

To our knowledge, although a lot of countries around the world are accelerating SARS-CoV-2 vaccine development, but no one is available until now for challenges of technical barriers.^[24]^ Furthermore, some COVID-19 patients could finally develop new problems, such as physical disability, cognitive impairment, psychological distress and symptoms of mental illness, increased vulnerability to recurrent infection further health deterioration.^[25]^ The SARS-CoV-2 might coexist with humans for a long time, just like influenza and chickenpox.^[26]^ A study conducted by Havard University projected that we may be confronted with another round of COVID-19 outbreak in winter.^[27]^ Therefore, it is critically important and urgent to find effective drugs or treatments and or preparation for the possible outbreak in the future. We also should keep an eye on prolonged mental illness of COVID-19 recovery patients.

TCM has long experience in treating pandemic and endemic diseases for almost thousand-of-years. In fact, the Chinese government attaches great importance to the role of TCM in the prevention and treatment of SARS-CoV-2, and for the first time that TCM as an independent therapy included in the guideline to diagnoses and treat COVID-19.^[28]^ Moreover, the total number of patients with diagnosed COVID-19 that treated by TCM is around 60,107.^[29]^ In 102 cases of the common symptoms treated with pure TCM, the clinical symptom disappearance time, the recovery time of body temperature, and the average length of stay in hospital were shortened by 2 days on average compared with control group. Both the rate of CT image improvement and the cure rate were increased by 22% and 33%. Also, TCM prevents 24% of the patients from common type to severe and critical forms.^[30]^

It is inspiring that lots of clinical trials are conducted and reported to evaluate the efficacy of TCM in treating SARS-CoV. However, most of them are poorly designed and lack of persuasion. In our multicenter prospective cohort study, *Baidu Jieduan Granule* we used is under the guidance of the theory “ Internal and External Relieving -Truncated Torsion ” and take the clinical characteristics of COVID-19 patients into consideration. By observing the changes in various indicators and imaging examination results during the disease, we seek to reveal the effectiveness and possible mechanism of *Baidu Jieduan Granule* in the treatment of COVID-19 using statistical analysis. Meantime, we expect to observe the safety and long-term mental health outcomes through follow-up.

This study has several limitations. First, we do not adopt randomized controlled clinical trials. Second, we only involve the common type of COVID-19 patients. Third, our follow-up time is not long enough.

## 4 Other information

### 4.1 Trial status

This protocol version is 2.0. This trial started on February 1, 2020, and is in the fellow-up phase at present.

### 4.2 Supplementary information

Additional file 1. SPIRIT checklist.

Additional file 2. APECHA II scoring system

Additional file 3. Liebowitz Social Anxiety Scale

Additional file 4. Depression, Anxiety and Stress Scale

Additional file 5. CRF

Additional file 6. Fund proofs

Additional file 7. Ethical proof

## Acknowledgments

We would like to express our appreciation to all medical centers, researchers, and patients, and thank for their cooperation.

## Author contributions

XXW and WL contributed equally to this study. BJF and SZ contribute to the study design, sponsored this research. Both XXW and WL finished draft and reversed the manuscript. QG and MC participated in the recruitment of patients and data statistics. CYL and WZ took part in data collection and coordinated the work of each center. WP, YQ together with JHC contributed to organizing the final data. All authors in this article have read the manuscript, and given their approval.

**Conceptualization:** Wei Lu, Yao Qu, Shuang Zhou.

**Data curation:** Quan Guo, Changya Liu, Yao Qu, Wei Peng, Shuang Zhou.

**Formal analysis:** Xinxin Wu, Quan Guo, Yao Qu.

**Funding acquisition:** Bangjiang Fang, Wei Lu.

**Investigation:** Wei Peng.

**Methodology:** Min Cao, Wen Zhang, Changya Liu, Jinhua Che.

**Project administration:** Bangjiang Fang, Shuang Zhou.

**Resources:** Min Cao, Wen Zhang.

**Software:** Wen Zhang, Changya Liu, Wei Peng, Jinhua Che.

**Validation:** Min Cao.

**Writing – original draft:** Xinxin Wu.

## Correction

In the first sentence of the article, “Since December 2020” has been corrected to “December 2019.”

## Supplementary Material

Supplemental Digital Content

## Supplementary Material

Supplemental Digital Content

## Supplementary Material

Supplemental Digital Content

## Supplementary Material

Supplemental Digital Content

## Supplementary Material

Supplemental Digital Content

## Supplementary Material

Supplemental Digital Content

## Supplementary Material

Supplemental Digital Content
