# Supplementary material for: Novel coronavirus pneumonia (COVID-19) combined with Chinese and Western medicine based on ”Internal and External Relieving -Truncated Torsion” strategy
Source: Medicine (Baltimore). 2020 Dec 18;99(51):e23874. doi: 10.1097/MD.0000000000023874 (PMC7748371; doi:10.1097/MD.0000000000023874)
Supplement: Supplemental Digital Content [file medi-99-e23874-s001.docx]

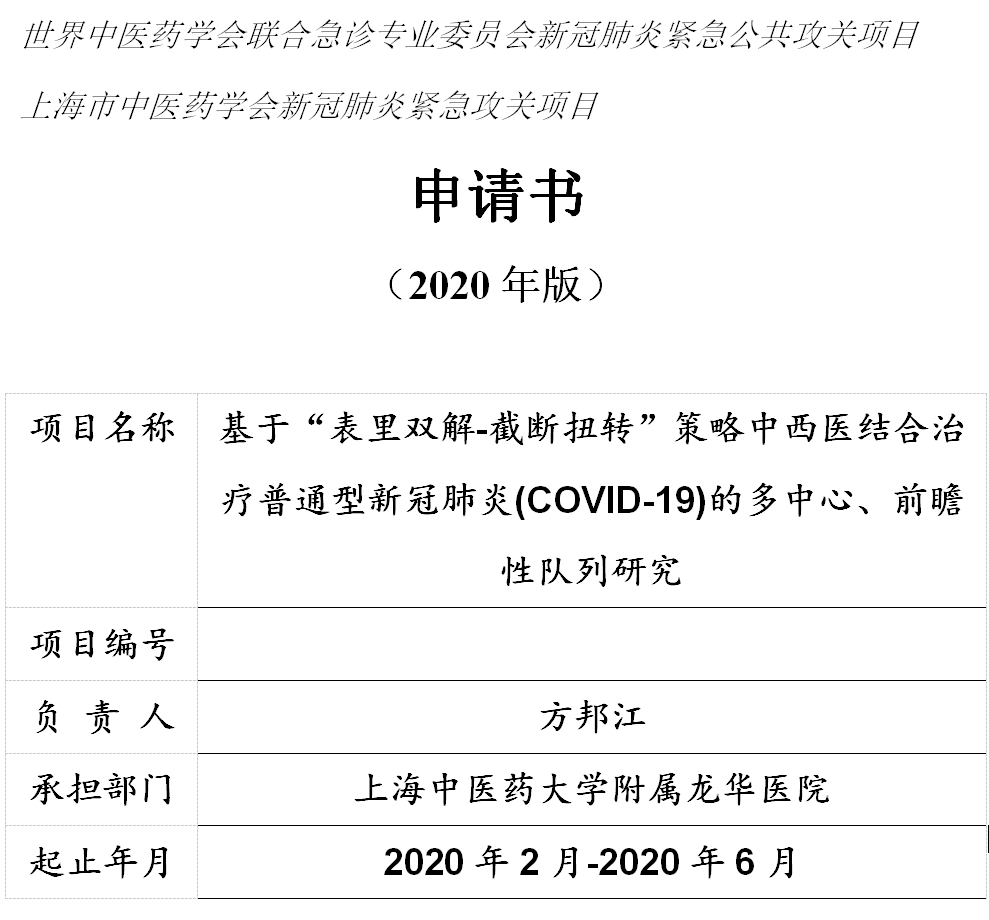


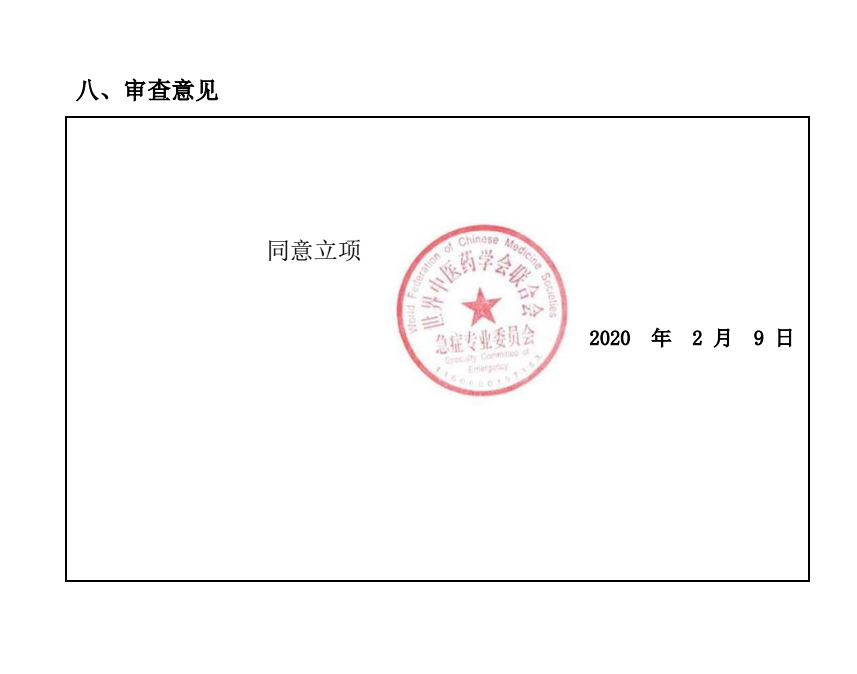


*Emergency Committee of the World Federation of Chinese Medicine Societies and Shanghai Society of Traditional Chinese Medicine and Shanghai Society of Traditional Chinese Medicine, Novel Coronavirus Pneumonia Emergency Tackling Key Project*

**The Project Plan**

**（2020 Version）**

| **Project name** | **Novel coronavirus pneumonia (COVID-19) combined with Chinese and Western medicine based on ''Internal and External Relieving -Truncated Torsion'' strategy** |
| --- | --- |
| **Project number** | SJZLJZ.N01 |
| **Project leaders** | Bangjiang Fang |
| **Primary sponsor** | LongHua Hospital Shanghai University of Traditional Chinese Medicine |
| **Project execute time** | 2020.02-2021.06 |


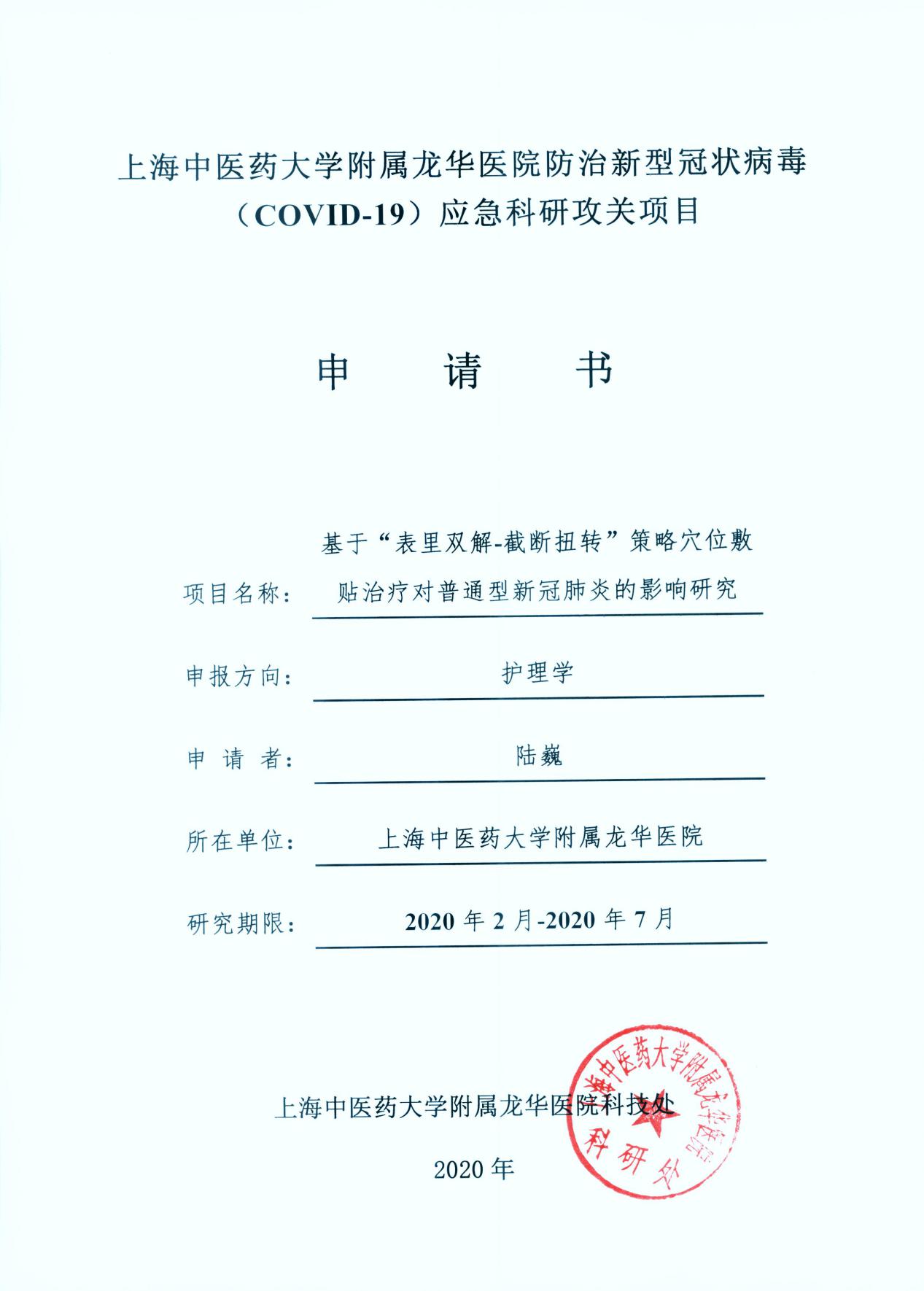


*Emergency research project for COVID-19 prevention and treatment int Longhua Hospital Shanghai University of Traditional Chinese Medicine*

**The Project Plan**

**（2020 Version）**

| **Project name** | Study on the influence of acupoint application therapy on the common type novel coronavirus disease (COVID-19) based on the strategy of "Exterior and Interior Solutions- Truncation and Reverse" |
| --- | --- |
| **Professional field** | Nursing |
| **Project leaders** | Wei Lu |
| **Primary sponsor** | LongHua Hospital Shanghai University of Traditional Chinese Medicine |
| **Project execute time** | 2020.02-2021.06 |


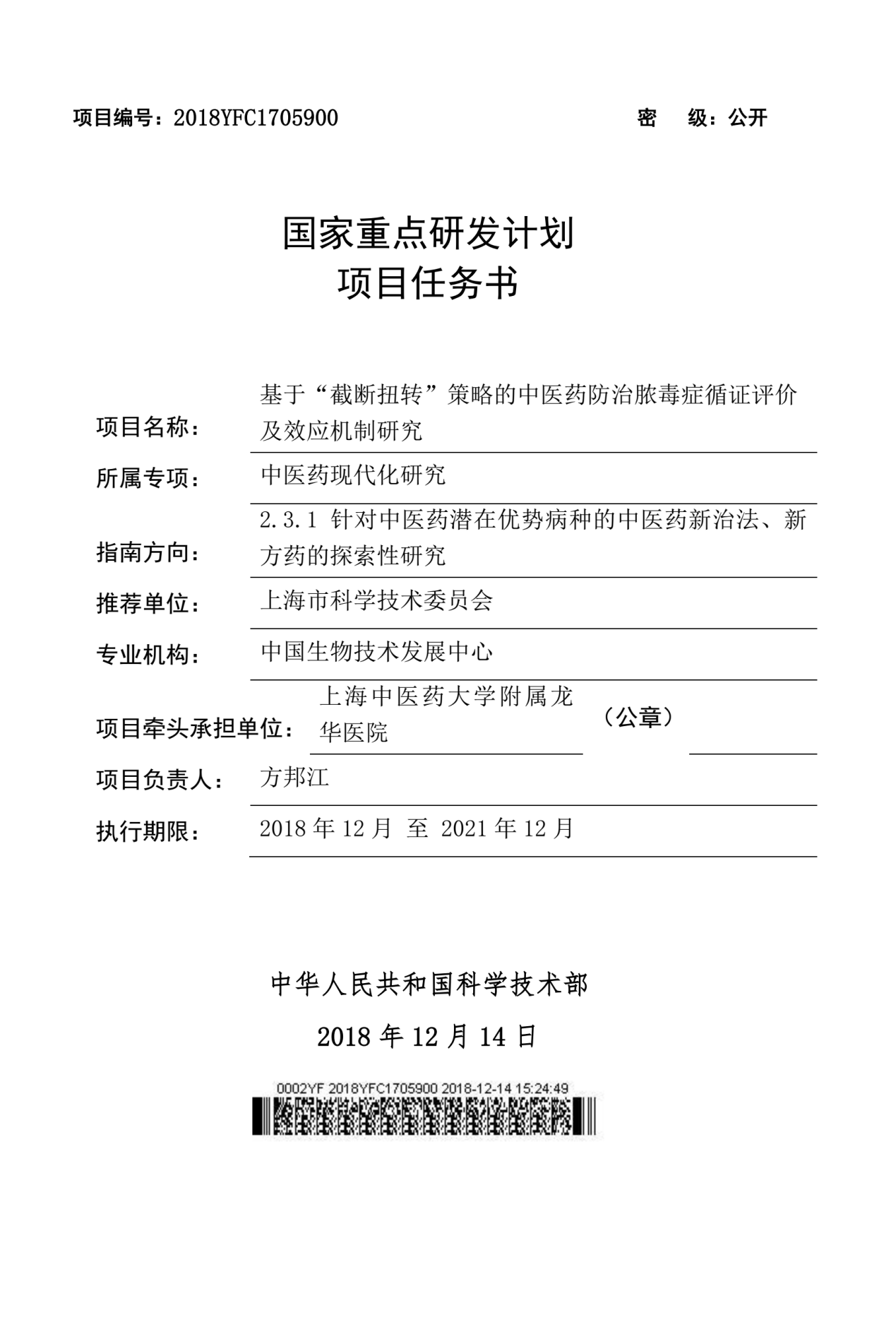


| **Project number：** | **2018YFC1705900** |  |
| --- | --- | --- |

National Key Research and Development Program

Project task document

| **Project name**： | Evidence-based evaluation and mechanism of TCM in the prevention and treatment of sepsis based on "Truncating and Reversing" method | |
| --- | --- | --- |
| **Project category：** | Research on modernization of traditional Chinese medicine | |
| **Programme Guidelines：** | 2.3.1 An exploratory study on the new therapy and formula of traditional Chinese medicine in treatment of potential preponderant diseases | |
| **Recommended units**： | Shanghai Science and Technology Committee | |
| **professional organizations**： | China biotechnology development center | |
| **Primary sponsor：** | | LongHua Hospital Shanghai University of Traditional Chinese Medicine |
| **Project leaders：** | Bangjiang Fang | |
| **execute time：** | 2019.01-2021.12 | |


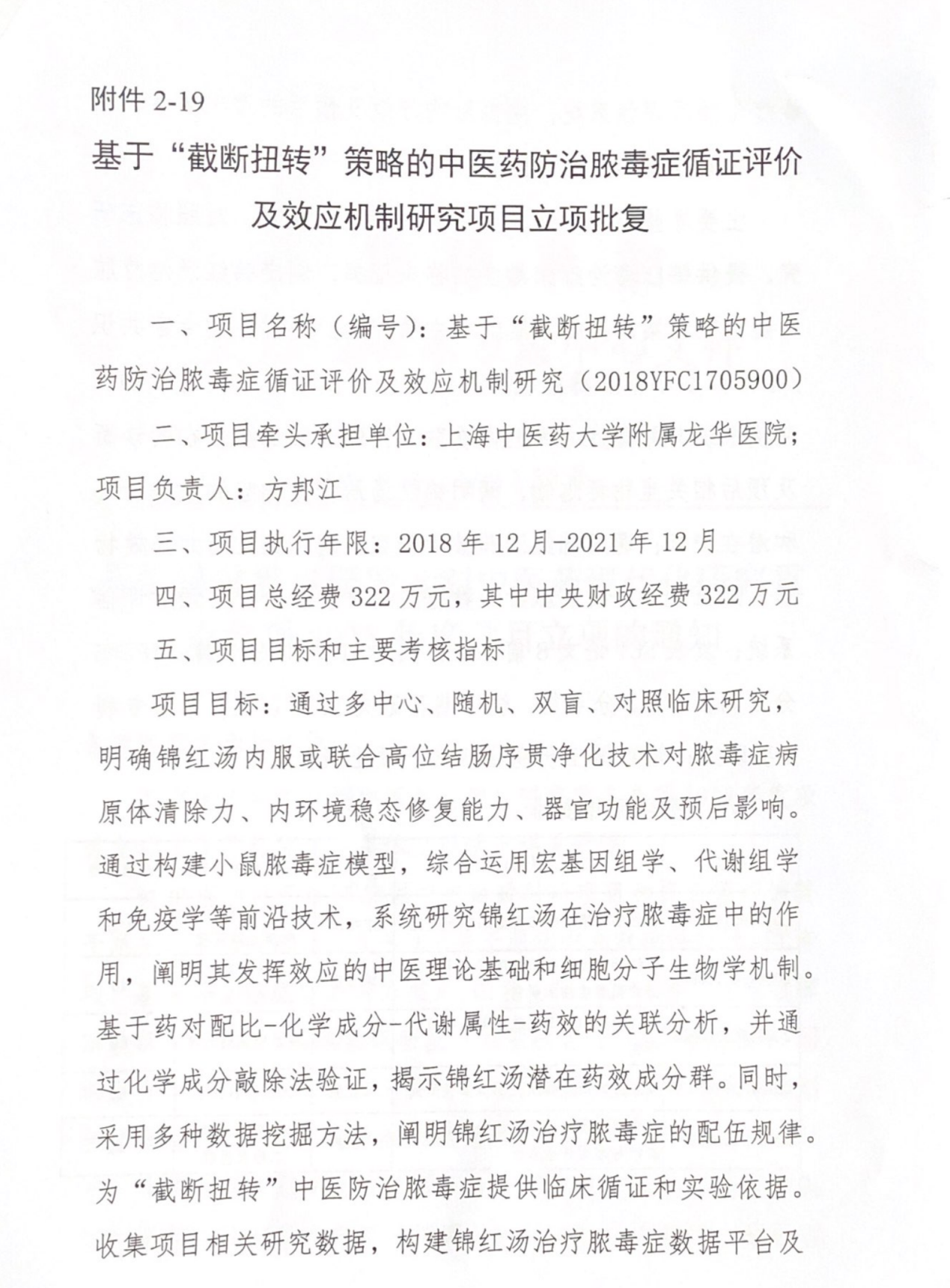


**The “evidence-based evaluation and mechanism of TCM in the prevention and treatment of sepsis based on Truncating and Reversing method” for the project has been approved**

1. The project name: evidence-based evaluation and mechanism of TCM in the prevention and treatment of sepsis based on Truncating and Reversing method
2. The undertaking unit of the projects: LongHua Hospital Shanghai University of Traditional Chinese Medicine
3. Execute time: 2018.12-2021.12
4. Evaluation indexes: Base on the fundamental therapeutic method of truncating and reversing, a multicenter, randomized, controlled clinical trial is performed combined with related preclinical research to evaluate the efficacy and safety and investigate mechanisms of Jinhong Decoction, which has been reported to have function of catharsis large intestine and eliminating evil, in prevention and treatment of sepsis.
