# Supplementary material for: Novel coronavirus pneumonia (COVID-19) combined with Chinese and Western medicine based on ”Internal and External Relieving -Truncated Torsion” strategy
Source: Medicine (Baltimore). 2020 Dec 18;99(51):e23874. doi: 10.1097/MD.0000000000023874 (PMC7748371; doi:10.1097/MD.0000000000023874)
Supplement: Supplemental Digital Content [file medi-99-e23874-s002.docx]

Ethical approval of Longhua Hospital Shanghai University of Traditional Chinese Medicine


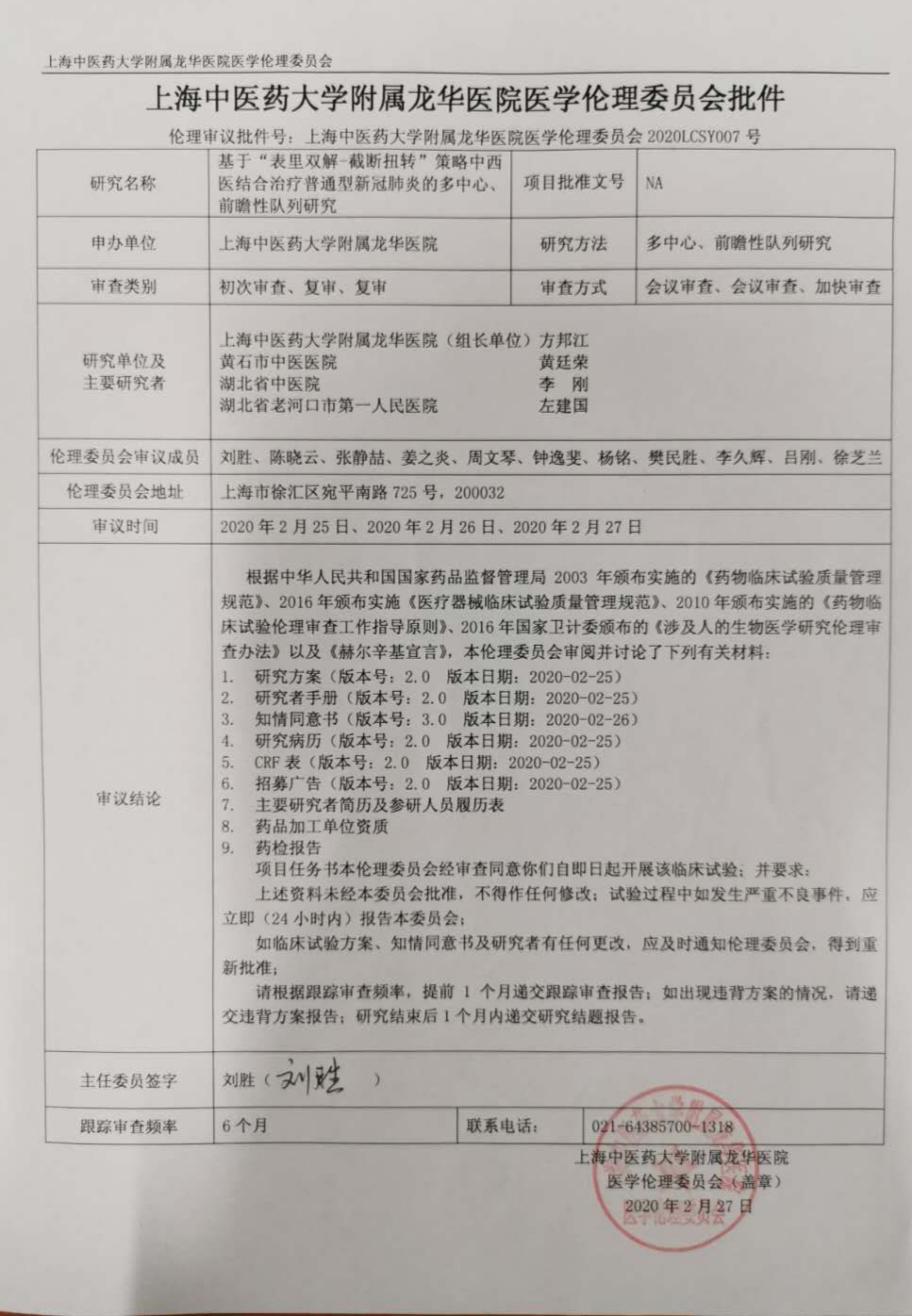


Ethics committee of Longhua Hospital Shanghai University of Traditional Chinese Medicine

**Approval of ethics committee of Longhua Hospital Shanghai University of Traditional Chinese Medicine**

**No:2020LCSY007**

| **Research Name** | A multicentre prospective cohort study for integrated Chinese and Western medicine in the treatment of the common type novel coronavirus disease (COVID-19) based on the “Exterior and Interior Solutions- Truncation and Reverse” | **Project approval number** | NA |
| --- | --- | --- | --- |
| **Organization** | Longhua Hospital Shanghai University of Traditional Chinese Medicine | **Research method** | multicenter, prospective, cohort study |
| **Review category** | initial review, reexamine | **Review method** | meeting review, speeding up review |
| **Research units and main researchers** | 1. Longhua Hospital Shanghai University of Traditional Chinese Medicine (leading medical center) Bangjiang Fang 2. Huangshi Hospital of TCM Yingrong Huang 3. Hubei Provincial Hospital of TCM Gang Li 4. LaoHeKou Hospital of TCM Jianguo Zuo | | |
| **Review members of the ethics committee** | Sheng Liu, Xiaoyun Chen, Jingji Zhang, Zhiyan Jiang, Wenqin Zhou, Yiwen Zhong, Ming Yang, Minsheng Fan, Jiuhui Li, Gang Lv, Zhilan Xu | | |
| **Address of ethics committee** | NO.725 Wanping South Road, Xuhui District, Shanghai | | |
| **Review time** | February 25, 2020 February 26, 2020 February 27, 2020 | | |
| **Review Results** | According to China Quality Management Standard of Drug Clinical Trials Issued and Implemented (2003), Quality Management Standard for Clinical trials of Medical Devices (2016), Guiding Principles for Ethical Review of Drug Clinical Trials (2010), Ethical Review of Biomedical Research for Designers (2016) and Helsinki Declaration, our ethics committee reviewed and discussed the following relevant materials:   1. research plan (version: 2.0, date: February 25, 2020) 2. investigator's brochure(version: 2.0, date: February 25, 2020) 3. [informed consent](http://dict.youdao.com/w/informed%20consent/#keyfrom=E2Ctranslation) (version: 2.0, date: February 25, 2020) 4. medical records (version: 2.0, date: February 25, 2020) 5. CRF form (version: 2.0, date: February 25, 2020) 6. recruitment advertisement (version: 2.0, date: February 25, 2020) 7. experience of major researchers 8. qualifications of pharmaceutical processing units 9. drug test report   The project task statement was approved and the team was agreed to carry out clinical trials.  The following must be observed:   1. the above information should not be modified without consent 2. any adverse events during the trial should be reported to the Ethics Committee immediately within 24 hours 3. submitting the follow-up review report one month in advance according to the review frequency 4. submitting a report on the violation ,in case of violation of the scheme 5. submitting the concluding report within 1 month when the study finished. | | |
| **Signature of chairman** | Sheng Liu | | |
| **Review frequency** | six months | **Phone number** | O21-64385700-1318 |
