# Supplementary material for: Novel coronavirus pneumonia (COVID-19) combined with Chinese and Western medicine based on ”Internal and External Relieving -Truncated Torsion” strategy
Source: Medicine (Baltimore). 2020 Dec 18;99(51):e23874. doi: 10.1097/MD.0000000000023874 (PMC7748371; doi:10.1097/MD.0000000000023874)
Supplement: Supplemental Digital Content [file medi-99-e23874-s004.docx]

**Liebowitz Social Anxiety Scale**

Name: Age: Date:

Fill out the following questionnaire with the most suitable answer listed below. Base you answers on you experience in the past week and, if you have complete the scale previously, be as consistent as possible in your perception of the situation described. Be sure to answer all items.

Fear or Anxiety: Avoidance:

0 = None 0 = Never (0%)

1 = Mild 1 = Occasionally (1—33%)

2 = Moderate 2 = Often (33—67%)

3 = Severe 3 = Usually (67—100%)

|  | **Fear or anxiety** | **Avoidance** |
| --- | --- | --- |
| 1. **Telephoning in public** - speaking on the telephone in a public place |  |  |
| 2. **Participating in small groups** – having a discussion with a few others |  |  |
| 3. **Eating in public places** – do you tremble or feel awkward handling food |  |  |
| 4. **Drinking with others in public places** – refers to any beverage including alcohol |  |  |
| 5. **Talking to people in authority** – for example, a boss or teacher |  |  |
| 6. **Acting, performing or giving a talk in front of an audience** – refers to a large audience |  |  |
| 7. **Going to a party** – an average party to which you may be invited; assume you know some but not all the people at the party |  |  |
| 8. **Working while being observed** – any type of work you might do including school work or housework |  |  |
| 9. **Writing while being observed** – for example, signing a check in a bank |  |  |
| 10. **Calling someone you don’t know very well** |  |  |
| 11. **Talking with people you don’t know very well** |  |  |
| 12. **Meeting strangers** – assume other are of the average importance to you |  |  |
| 13. **Urinating in a public bathroom** – assume that others are sometimes present, as might normally be expected |  |  |
| 14. **Entering a room when others are already seated** – refers to a small group, and nobody has to move seats for you |  |  |
| 15. **Being the center of attention** – telling a story to a group of people |  |  |
| 16. **Speaking up at a meeting** – speaking from your seat in a small meeting or standing up in place in a large meeting |  |  |
| 17. **Taking a written test** |  |  |
| 18. **Expressing appropriate disagreement or disapproval to people**  **you don’t know very well** |  |  |
| 19. **Looking at people you don’t know very well in the eyes** – refers to appropriate eye contact |  |  |
| 20**. Giving a report to a group** – refers to an oral report to a small group |  |  |
| 21. **Trying to pick up someone** – refers to a single person attempting to initiate a relationship with a stranger |  |  |
| 22. **Returning goods to a store where returns are normally accepted** |  |  |
| 23. **Giving an average party** |  |  |
| 24. **Resisting a high pressure salesperson** – avoidance refers to listening to a salesperson too long |  |  |

The scoring scale: 55-65 Moderate social phobia; 65-80 Marked social phobia; 80-95 Severe social phobia; Greater than 95 - Very severe social phobia
