# Supplementary material for: Novel coronavirus pneumonia (COVID-19) combined with Chinese and Western medicine based on ”Internal and External Relieving -Truncated Torsion” strategy
Source: Medicine (Baltimore). 2020 Dec 18;99(51):e23874. doi: 10.1097/MD.0000000000023874 (PMC7748371; doi:10.1097/MD.0000000000023874)
Supplement: Supplemental Digital Content [file medi-99-e23874-s005.docx]

**Depression, Anxiety and Stress Scale (DASS21)**

For each statement below, please circle the number in the column that best represents how you have been feeling in the last week.

| **Statement** | **Did not apply to me at all** | **Applied to me to some degree or**  **some of the time** | **Applied to me a considerable degree or a**  **good part of the time** | **Applied to me very much or**  **most of the time** |
| --- | --- | --- | --- | --- |
| 1. I found it hard to wind down | 0 | 1 | 2 | 3 |
| 2. I was aware of dryness of my mouth | 0 | 1 | 2 | 3 |
| 3. I couldn't seem to experience any positive feeling at all | 0 | 1 | 2 | 3 |
| 4. I experienced breathing difficulty (eg, excessively rapid breathing, breathlessness in the absence of physical exertion) | 0 | 1 | 2 | 3 |
| 5. I found it difficult to work up the initiative to do things | 0 | 1 | 2 | 3 |
| 6. I tended to over-react to situations | 0 | 1 | 2 | 3 |
| 7. I experienced trembling (eg, in the hands) | 0 | 1 | 2 | 3 |
| 8. I felt that I was using a lot of nervous energy | 0 | 1 | 2 | 3 |
| 9. I was worried about situations in which I might panic and make a fool of myself | 0 | 1 | 2 | 3 |
| 10. I felt that I had nothing to look forward to | 0 | 1 | 2 | 3 |
| 11. I found myself getting agitated | 0 | 1 | 2 | 3 |
| 12. I found it difficult to relax | 0 | 1 | 2 | 3 |
| 13. I felt down-hearted and blue | 0 | 1 | 2 | 3 |
| 14. I was intolerant of anything that kept me from getting on with what I was doing | 0 | 1 | 2 | 3 |
| 15. I felt I was close to panic | 0 | 1 | 2 | 3 |
| 16. I was unable to become enthusiastic about anything. | 0 | 1 | 2 | 3 |
| 17. I felt I wasn't worth much as a person | 0 | 1 | 2 | 3 |
| 18. I felt that I was rather touchy | 0 | 1 | 2 | 3 |
| 19. I was aware of the action of my heart in the absence of physical exertion (eg, sense of heart rate increase, heart missing a beat) | 0 | 1 | 2 | 3 |
| 20. I felt scared without any good reason. | 0 | 1 | 2 | 3 |
| 21. I felt that life was meaningless | 0 | 1 | 2 | 3 |

Lovibond, S.H. & Lovibond, P.F. (1995). Manual for the Depression Anxiety Stress Scales. (2nd. Ed.) Sydney: Psychology Foundation

**DASS21 SCORING**

1. For questions numbered 3, 5, 10, 13, 16, 17, 21 add up the numbers circled then multiply that number by 2 and enter it here:
2. For questions numbered 2, 4, 7, 9, 15, 19, 20 add up the numbers circled then multiply that number by 2 and enter it here:
3. For questions numbered 1, 6, 8, 11, 12, 14, 18 add up the numbers circled then multiply that number by 2 and enter it here:

Refer to the chart below and for each numbered question above, refer to the same number in the table below to determine how mild or serious each condition may be.

| **Rating** | **Depression**  **#1** | **Anxiety**  **#2** | **Stress**  **#3** |
| --- | --- | --- | --- |
| Normal | 0-9 | 0-7 | 0-14 |
| Mild | 10-13 | 8-9 | 15-18 |
| Moderate | 14-20 | 10-14 | 19-25 |
| Severe | 21-27 | 15-19 | 26-33 |
| Extremely Severe | 28+ | 20+ | 37+ |

Provided to you by [Depression-Test.net](http://www.depression-test.net/) for educational purposes only. If there is an indication that you might be depressed, please check out the site for additional information, tools and support.

If there is an indication that It might be serious then please see the help of a mental health professional.
