# Supplementary material for: Novel coronavirus pneumonia (COVID-19) combined with Chinese and Western medicine based on ”Internal and External Relieving -Truncated Torsion” strategy
Source: Medicine (Baltimore). 2020 Dec 18;99(51):e23874. doi: 10.1097/MD.0000000000023874 (PMC7748371; doi:10.1097/MD.0000000000023874)
Supplement: Supplemental Digital Content [file medi-99-e23874-s006.docx]

**Case report form**

| **Research center number**  **□□** | **Patients number**  **□□□□□** | **Name**  **□□□□** | **Date of enrollment**  **□□□□-□□-□□** |
| --- | --- | --- | --- |

| **Inclusion criteria** | **yes** | **No** |
| --- | --- | --- |
| 1. COVID-19 conformed |  |  |
| - SARS-CoV-2 nucleic acid testing of the respiratory specimens shows positive by means of real-time reverse transcription polymerase chain reaction (RT-PCR) assay. | □ | □ |
| - The tested virus gene is highly homologous to SARS-CoV-2. | □ | □ |
| - Serum SARS-CoV-2 lgM and lgG antibody are positive. | □ | □ |
| - Serum SARS-CoV-2 IgG antibody turned positive or the serum novel coronavirus specific IgG antibody in recovery phase was 4 times or more higher than that in acute phase. |  |  |
| 1. 18 years old ≤ age ≤ 80 years old. | □ | □ |
| 1. Informed consent, participate in this study voluntarily. | □ | □ |

| **Exclusion criteria** | **Yes** | **No** |
| --- | --- | --- |
| 1. Patients with allergy and allergy to *Baidu Jieduan Formula.* | □ | □ |
| 1. Pregnant and lactating women. | □ | □ |
| 1. Patients with obstructive pneumonia, pulmonary interstitial fibrosis, pulmonary alveolar   proteinosis and allergic alveolitis caused by lung tumor | □ | □ |
| 1. Patients with malignant tumor, liver cirrhosis, chronic renal failure (uremic stage), hematological disease, HIV and other serious basic diseases. | □ | □ |
| 1. Patients who were treated with hormones and Immunosuppressant for a long time. | □ | □ |
| 1. Patients who suffer from severe mental illness or are unable to cooperate with this experiment. | □ | □ |

| **Patient withdrawal** | **yes** | **No** |
| --- | --- | --- |
| 1. Subjects experiencing serious adverse events at any time during the treatment period. | □ | □ |
| 1. Give up the test midway. | □ | □ |

1. **Basic information of patients**

| **Gender** | □male □female | **Nation** |  | | |
| --- | --- | --- | --- | --- | --- |
| **Date of birth** | □□□□-□□-□□ | **Height** | □□□cm | **Weight** | □□□kg |
| **Date of diagnosis** | □□□□-□□-□□ | **Date of admission** | □□□□-□□-□□ | | |
| **Underlying disease** | □diabetes □hypertension □coronary heart disease □cancer □heart failure  □Cerebrovascular diseases □other disease  □肾脏疾病 □肝脏疾病 | | | | |
| **Grouping** | □experimental group：*Baidu Jieduan Formula* together with standard Western medicine for 14 days.  □control group： standard Western medicine for 14 days. | | | | |

| **History of drug allergy** | □No □Yes Name of allergic drug:: _________________________________ |
| --- | --- |
| **Other allergic history** | □No □Yes Allergen name: _______________________________________ |
| **Smoking history** | □No □Yes Smoking index (number of cigarettes per day×years of smoking)： |
| **History of alcoholism** | □No □Yes |

1. **Chest computed tomograghy examination record and score**

| **D1** | Description and score： |
| --- | --- |
| **D7** | Description and score： |
| **D14** | Description and score： |

1. **Vital signs (1-7 day)**

|  | **D1** | **D2** | **D3** | **D4** | **D5** | **D6** | **D7** |
| --- | --- | --- | --- | --- | --- | --- | --- |
| **Temperature (℃)** |  |  |  |  |  |  |  |
| **Heart rate (time/min)** |  |  |  |  |  |  |  |
| **Respiratory rate (time/min)** |  |  |  |  |  |  |  |
| **Systolic pressure（mmHg）** |  |  |  |  |  |  |  |
| **Diastolic pressure（mmHg）** |  |  |  |  |  |  |  |
| **Blood oxygen saturation (SaO2)** |  |  |  |  |  |  |  |

1. **Vital signs (8-14 day)**

|  | **D1** | **D2** | **D3** | **D4** | **D5** | **D6** | **D7** |
| --- | --- | --- | --- | --- | --- | --- | --- |
| **Temperature (℃)** |  |  |  |  |  |  |  |
| **Heart rate (time/min)** |  |  |  |  |  |  |  |
| **Respiratory rate (time/min)** |  |  |  |  |  |  |  |
| **Systolic pressure（mmHg）** |  |  |  |  |  |  |  |
| **Diastolic pressure（mmHg）** |  |  |  |  |  |  |  |
| **Blood oxygen saturation (SaO2)** |  |  |  |  |  |  |  |

1. **Inspection results**

|  | **D1** | **D3** | **D5** | **D7** | **D14** |
| --- | --- | --- | --- | --- | --- |
| **Red blood cell count (RBC, 10^12^/L)** |  |  |  |  |  |
| **Red blood cell count (WBC, 10^9^/L)** |  |  |  |  |  |
| **Percentage of lymphocytes（%）** |  |  |  |  |  |
| **Platelet count**  **(PLT, 10^9^/L)** |  |  |  |  |  |
| **Alanine aminotransferase (ALT, U/L)** |  |  |  |  |  |
| **Aspartate aminotransferase**  **(AST, U/L)** |  |  |  |  |  |
| **Serum total bilirubin**  **(TB, μmol/L)** |  |  |  |  |  |
| **Blood urea nitrogen**  **( BUN, mmol/L)** |  |  |  |  |  |
| **Serum creatinine**  (**Cr, μmol/L)** |  |  |  |  |  |
| **Fibrinogen**  **(Fg, mg/dl）** |  |  |  |  |  |
| **Prothrombin time (PT, S)** |  |  |  |  |  |
| **Activated partial thromboplastin time**  (**APTT, S)** |  |  |  |  |  |
| **D-Dimer**  **(μg/mL)** |  |  |  |  |  |
| 1. **reactive protein,**   **(CRO, mg/dl)** |  |  |  |  |  |
| **Procalcitonin**  **(PCT, ug/L)** |  |  |  |  |  |

|  | **D1** | **D3** | **D5** | **D7** | **D14** |
| --- | --- | --- | --- | --- | --- |
| **Tumor necrosis factor**  **（TNF-α, g/ml）** |  |  |  |  |  |
| **Interleukin-6**  **(IL-6, pg/ml)** |  |  |  |  |  |
| **Interleukin-10**  **(IL-10, pg/ml)** |  |  |  |  |  |

1. **Clinical outcomes after 14 days observation**

| **Clinical outcomes** | □Recovery □Condition improved □Condition worsen □death |
| --- | --- |
| **🞏Discharged** | Date：□□□□-□□-□□ |
| **🞏Death** | Date：□□□□-□□-□□ |
|  | Cause of death:（Choosing the main one）  □ Shock □ Respiratory failure □ Heart failure  □ Renal failure □ Liver failure □ Other reasons: |

1. **Adverse event (AE) recording**

| **Name of AE** | **Description of AE** | **Date** | **Solution** | **Outcomes** | **Relationship with research drug** |
| --- | --- | --- | --- | --- | --- |
|  |  |  | □Continuing to use  □Dose reduction  □Suspended medication  □Discontinued | □Exacerbation  □ Remission (Date: )  □No improvement  □Recovery (Date )  □Sequela: □Yes □No | □Definitely relevant  □Probably related  □May be relevant  □May be irrelevant  □Definitely irrelevant  □Unable to evaluate |
|  |  |  | □Continuing to use  □Dose reduction  □Suspended medication  □Discontinued | □Exacerbation  □Remission (Date: )  □No improvement  □Recovery (Date )  □Sequela: □Yes □No | □Definitely relevant  □probably related  □May be relevant  □May be irrelevant  □Definitely irrelevant  □Unable to evaluate |
|  |  |  | □Continuing to use  □Dose reduction  □Suspended medication  □Discontinued | □Exacerbation  □ Remission (Date: )  □No improvement  □Recovery (Date )  □Sequela: □Yes □No | □Definitely relevant  □probably related  □May be relevant  □May be irrelevant  □Definitely irrelevant  □Unable to evaluate |

1. **Evaluation criteria for correlation of AE**

| **Result of judgment** | **Description of AE** | **Reasonable time** | **Whether the symptoms disappear after stopping or reducing the dose** | **Symptoms appeared after repeated administration** | **The progress of combined medication affects other treatments** |
| --- | --- | --- | --- | --- | --- |
| **Definitely relevant** |  |  |  |  |  |
| **Probably related** |  |  |  |  |  |
| **May be relevant** |  |  |  |  |  |
| **May be irrelevant** |  |  |  |  |  |
| **Definitely irrelevant** | Non compliance with appeal items | | | | |
| **Unable to evaluate** | Insufficient evidence to evaluate | | | | |

**Completed by：**
